# Oropharyngeal Cancer Epidemic and Human Papillomavirus

**DOI:** 10.3201/eid1611.100452

**Published:** 2010-11

**Authors:** Torbjörn Ramqvist, Tina Dalianis

**Affiliations:** Author affiliations: Karolinska Institutet, Stockholm, Sweden (T. Ramqvist, T. Dalianis);; Swedish Institute for Infectious Disease Control, Solna, Sweden (T. Dalianis)

**Keywords:** Viruses, HPV, papillomavirus, oropharyngeal cancer, cancer, tonsillar cancer, base of tongue cancer, head and neck cancer, synopsis

## Abstract

A growing body of research shows that human papillomavirus (HPV) is a common and increasing cause of oropharyngeal squamous cell carcinoma (OSCC). Thus, the International Agency for Research against Cancer has acknowledged HPV as a risk factor for OSCC, in addition to smoking and alcohol consumption. Recently, in Finland, the United Kingdom, the Netherlands, the United States, and Sweden, incidence of OSCC has increased, and an increase in the proportion of HPV-positive tumors was noted. On the basis of these data and reports indicating that patients with HPV-positive cancer have their first sexual experience at a young age and have multiple partners, we postulate that increased incidence of OSCC in the United States and some countries in northern Europe is because of a new, primarily sexually transmitted HPV epidemic. We also suggest that individualized treatment modalities and preventive vaccination should be further explored.

## Abstract 2

Patients with HPV-positive cancer were young and lacked traditional risk factors.

Oropharyngeal Cancer Epidemic and HPV

In many countries, vaccines against some human papillomavirus (HPV) types are now administered to girls and young women with the goal of protecting them against HPV-induced cervical cancer (*1**,**2*). The introduction of HPV vaccines has also drawn more attention to the fact that HPV is associated not only with cervical cancer and genital warts but also with other tumors, such as head neck and anogenital cancers (*3*). We focus on the role of HPV in the increased incidence of oropharyngeal squamous cell carcinoma (OSCC), the head and neck cancer in which HPV is most commonly found (*4*).

Head and neck cancer most commonly is of the squamous cell carcinoma type (HNSCC) and includes cancers of the oral cavity, oropharynx, hypopharynx, larynx, sinonasal tract, and nasopharynx. HNSCC is the sixth most common type of cancer in the world; almost 600,000 cases are reported annually, and of these, ≈10% (or more for some geographic locations) are OSCC (*5*). Globally, the incidence and localization of HNSCC varies widely. It is the most common form of cancer in India, and incidence is higher in countries in Latin America than in the United States and northern Europe. In addition, men are generally more often affected than women. Smoking, alcohol consumption, and betel chewing are traditional risk factors for HNSCC and OSCC (*6*). However, during the past decade several reports have documented HPV in OSCC (*7**–**9*). HPV infection, with dominance of HPV16 infection, has therefore been acknowledged by the International Agency for Research against Cancer as a risk factor for OSCC (*10*). Moreover, there are accumulating reports from many countries that the incidence of OSCC is increasing. We suggest that this increase is caused by a slow epidemic of HPV infection–induced OSCC.

## OSCC

Tonsillar cancer is the most common OSCC, followed by base of tongue cancer. Together, these 2 cancers account for 90% of all OSCCs (*6**,**9*). Patients usually do not seek counseling until the tumors are large because small tumors cause little distress and may not be noticed by the patient. Curative treatment implies surgery, radiotherapy, and chemotherapy; the goal is to cause as little functional and cosmetic damage as possible (*6**,**9*). If a cure cannot be obtained, palliative therapy is given to treat pain and discomfort. Similar to HNSCC, in general, survival rates for patients with OSCC are poor. Patients with OSCC have an overall 5-year survival rate of ≈25% (*6**,**9*). Furthermore, even when standardized treatment is used and tumors are at the same stage and have similar histologic features, it is difficult to predict the outcome. Several reports now describe the incidence of OSCC as increasing and indicate that HPV-positive OSCC has a better clinical outcome than HPV-negative OSCC (*7**–**9**,**11**–**19*). Thus, predictive and prognostic markers would be of clinical value for prevention and treatment of OSCC.

## HPV

There are >100 HPV types, some found in skin warts and others in mucous tissues, and the association of different HPV types with cervical, some anogenital, and head and neck cancers is well established (*3*). The 8-kb, double-stranded, circular DNA HPV genome, enclosed in a 52–55 nm viral capsid, codes for the L1 and L2 viral capsid proteins and for the E1–E2 and E4–E7 proteins, which play major roles in gene regulation, replication, pathogenesis, and transformation (*3*). In high-risk HPVs (i.e., those that are more likely to cause lesions that may develop into cancer [www.cancer.gov/cancertopics/factsheet/Risk/HPV]), E6 and E7 deregulate cell cycle control by E6 binding and degradation of p53, and E7 binds and inhibits the function of the retinoblastoma protein (Rb) (*3*). The L1 protein can self-assemble into virus-like particles, which form the basis of both currently approved vaccines against HPV infection (*1**–**3*).

## HPV and Methods for Detection in OSCC

During the past few decades, HPV DNA has been detected in ≈25% of HNSCCs overall, but especially in OSCC, for which 45%–100% cases were reported to be HPV positive (*7**–**9**,**11**–**19*). The latter variation may depend on OSCC location, the type of specimens available, the techniques used for testing, and the time period and country from which the sample material was obtained (*7**–**9**,**11**–**19*).

Analysis of HPV DNA was (and still is) performed primarily by using formalin-fixed, paraffin-embedded tissue, in which the DNA can be partially degraded. It is now widely accepted that it is easier to detect longer HPV DNA fragments in fresh or fresh-frozen material, although newer techniques are more sensitive. Many early studies during the 1980s were based on Southern blot techniques or in situ hybridization for detection of HPV.

Since the 1990s, virology laboratories used PCR for detection of HPV DNA (*20**–**23*). Screening for HPV was initially performed by using general PCR primers for HPV, which enabled detection of several HPV types (*21**–**23*). PCR of a control cellular gene was used to assess the DNA quality of samples. These techniques are robust and are still used but need additional methods for HPV typing. There are now many other methods that directly determine the presence of several different HPV types. The Food and Drug Administration–approved Hybrid Capture II (Digene Corporation, Gaithersburg, MD, USA) detects 5 low-risk and 13 high-risk HPV types and uses the fact that HPV DNA hybridizes with synthetic RNA probes complementary to DNA sequences from specific HPV types (*20*). An assay used in several studies, the Roche (Basel, Switzerland) linear array HPV Genotyping Test, detects 37 HPV types and is based on a method developed by Gravitt et al. (*24*). In this method, HPV PCR products are hybridized to a linear array of type specific probes. Recently, Schmitt et al developed a sensitive bead-based multiplex method, originally set up for 22 different HPV types but later expanded, in which HPV PCR products are coupled to type-specific probes on beads and analyzed by using Luminex (*25*).

To assay for biologic activity of HPV in tumors, analysis of E6 and E7 expression by detecting E6 and E7 mRNA by reverse transcription followed by real-time PCR is also often performed (*13*). In pathology departments, HPV screening is often conducted by in situ hybridization, and in some instances p16 immunohistochemical analysis is used as a substitute to assay for biologically active HPV because there is a correlation between the presence of HPV and overexpression of p16 (*26**,**27*).

## HPV in OSCC

When HPV in OSCC became more obvious, several studies concentrated on characterizing HPV-positive OSCC (*4**,**7**–**9**,**11**–**19*). HPV type 16 was highly prevalent (≈90%) in OSCC in all studies; other HPV types (e.g., HPV-31, -33, -58, -59, -62, and -72) were less common, and HPV was demonstrated to be episomal or integrated into the cellular genome (*14**,**28*).

In several studies, E6 and E7 expression in OSCC were shown, suggesting that HPV was actively involved in the etiology of the tumors (*13*). In addition, the association of p16 overexpression with HPV was a further indicator of active E7 because of E7-induced cell cycle activation and upregulation of p16 by inactivating the Rb pathway (*3**,**26**,**27*). HPV-positive tumors were also less likely to have mutated p53 (*7*) and were more frequently aneuploid and less differentiated than HPV-negative tumors (*29*). Furthermore, comparative genomic hybridization indicated that HPV-positive tonsillar cancer, in contrast to HPV-negative cancer, often showed chromosome 3q amplification similar to that in HPV-positive cervical and vulvar cancer, which further supports the oncogenic role of HPV in OSCC (*30*).

**Figure 1:**
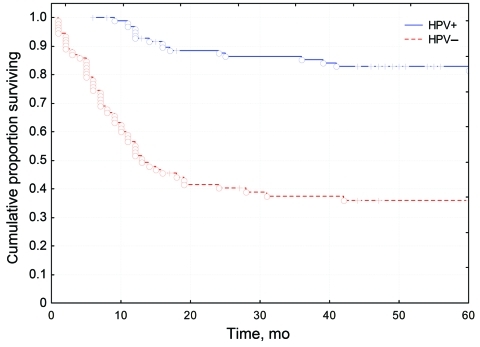
Survival rates for patients with human papillomavirus (HPV)–positive tonsillar cancer compared with those for patients with HPV-negative cancer. Circles indicate patients who died of tonsillar cancer during the follow-up period; plus signs indicate patients who were removed from the study for other reasons (e.g., died from a cause other than tonsillar cancer, left the country); p<0.0005. Data from Lindquist et al. (*13*), with permission of Elsevier (www.elsevier.com).

It was also observed that patients with HPV-positive OSCC were younger and lacked the traditional risk factors of smoking and alcohol consumption (*7**–**9**,**13**,**17*). Moreover, a major feature, noted in several studies, was that HPV was a favorable prognostic factor for clinical outcome of OSCC, as demonstrated in Figure 1 (*7**–**9**,**11**–**19*). This finding was independent of tumor stage, age, gender, grade of differentiation, p53 immunohistochemical results, or DNA ploidy (*7**–**9*). However, Lindquist et al. (*13*) observed that patients with HPV-positive tonsillar cancer who had never smoked had a better prognosis than those who were smokers, and this observation was recently confirmed by Ang et al. (*16*). The reasons for this finding are most likely complex and should be investigated further. One could speculate, for example, that HPV induces an immune response and that smoking abrogates this response. A different option is that smoking and HPV in combination induce a different category of tumors and that smoking induces additional genetic alterations in these tumors, as was also suggested by Ang et al. (*16*).

In general, the prognostic value of HPV status is for OSCC and not for HNSCC. In some studies with conflicting findings, the prognostic value of HPV was determined for all HNSCC anatomic sites (*9*). However, because there are differences in survival rates and presence of HPV at different locations, even for OSCC, studies should be performed per location.

In summary, the accumulated data suggest different entities of OSCC, where some primarily depend on smoking and alcohol and others on HPV infection. It is also likely that there are combined etiologies. Nevertheless, patients with HPV-positive OSCC consistently have a better prognosis (*7**–**9**,**11**–**19*).

## An HPV-induced Epidemic of OSCC

We suggest the increased incidence of OSCC depends on HPV infection and results in an increased proportion of HPV-positive OSCCs. During the past decades, studies from the United States, Finland, Sweden, the Netherlands, the United Kingdom, and Scotland showed an increase in the incidence of OSCC, tonsillar cancer, and base of tongue cancer (*31**–**36*). In addition, during the past 10 years, an increase in the proportion of HPV-positive OSCC has been reported, and we speculate that this is not caused simply by use of more sensitive diagnostic techniques. In many of these studies the same assay was used when studying OSCC over time (*12**,**18**,**19*). Furthermore, the general PCR amplifiability of the DNA from the older tested samples was also validated.

Using the Swedish Cancer Registry, which covers basically all cancer cases in Sweden for 1970–2002, we disclosed a 2.8-fold increase (2.6-fold for men and 3.5-fold for women) in the incidence of tonsillar cancer in the Stockholm area (*12*), where 25%–30% of all patients in Sweden with tonsillar cancer are treated. In parallel, we examined all 237 available samples from the 515 patients with tonsillar cancer in Stockholm during the same period and found a 2.9-fold increase in the proportion of HPV-positive tonsillar cancer from 23% to 68% (*12*). We thus suggested HPV infection played a role in the increase of this disease (*12*).

**Figure 2:**
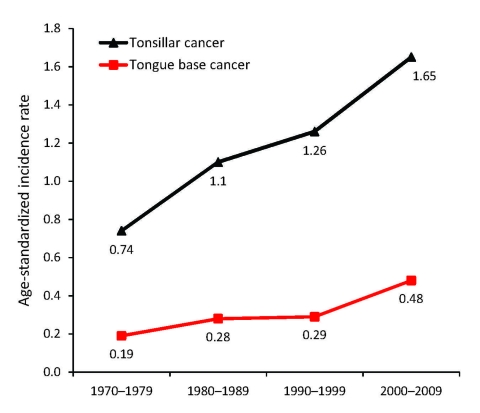
Age-standardized incidence of tonsillar and base of tongue cancers, Stockholm, Sweden, 1970–2006.

**Figure 3:**
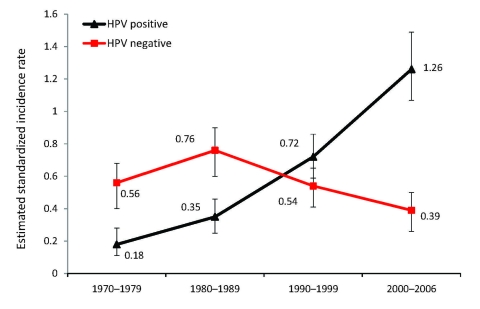
Estimated age-standardized incidence of human papillomavirus (HPV)–positive and HPV-negative tonsillar cancer squamous cell carcinoma cases per 100,000 person-years, Stockholm, Sweden, 1970–2006. Error bars indicate 95% confidence intervals. Data from Näsman et al. (*13*), with permission of John Wiley and Sons (www.interscience.wiley.com).

In continuation of the above study, we followed the incidence of tonsillar and base of tongue cancer in Stockholm in the Swedish Cancer Registry and demonstrated a substantial increase for both tumor types during 1970–2006, as shown in Figure 2 (*18**,**19**,**34*). We then performed a follow-up study in the Stockholm area of the prevalence of HPV in tonsillar cancer during 2003–2007; using the Swedish Cancer Registry, we identified 120 patients (*18*). Using the same methods as in the first study, we found that the proportion of HPV-positive cancers in the 98 available pretreatment biopsy specimens had significantly increased both from 1970 through 2007 (p<0.0001) and from 2000 through 2007 (p<0.01). During the last 2 years of the study (2006–2007), 93% of all tonsillar cancer was HPV positive. Moreover, the incidence of HPV-positive tumors almost doubled each decade during 1970–2007, indicating a 7-fold increase over the whole period; in parallel, a decline of HPV-negative tumors was observed (Figure 3).

Shortly afterwards, we demonstrated that the prevalence of HPV-positive base of tongue cancer also had increased in the Stockholm area during 1998–2007 (*19*). When we analyzed 95 pretreatment biopsy specimens from base of tongue SCC from the 109 patients reported during 1998–2007 in the Swedish Cancer Registry in a similar way as above, we found an increase in the proportion of HPV-positive tumors from 54% in 1998–1999 to 84% in 2006–2007.

The strength of the above studies is that the Swedish Cancer Registry covers all cancer cases in Sweden and that we have analyzed all available pretreatment diagnostic biopsy specimens for HPV. The limitation of our study is that Sweden is a small country with only 9 million inhabitants.

Covering almost the same time period as above, Chaturvedi et al. reported an increase in the proportion of potentially HPV-related OSCC in the United States (*35*). However, the previous year, Sturgis and Cinciripini had already proposed a possible emerging epidemic of HPV-associated cancers (*11*). More recently, Marur et al. reviewed many studies, further supporting an increase in OSCC (*17*). Notably, it was also suggested that the increase in OSCC occurs mainly in men (*17*). However, using the Swedish Cancer Registry, in the Stockholm area, Hammarstedt et al. observed also an increase of OSCC in women (*12*). It is necessary to acknowledge that the numbers of women with OSCC are more limited and that it could be more difficult to identify major changes in this group.

The possible causes for this increase have been discussed extensively and have focused on changes in sexual patterns, such as increased oral sex or increasing numbers of sex partners. A significant association has been shown between HPV-positive tonsillar cancer and early initial sex or number of oral or vaginal sex partners (*37*).

Furthermore, in a recent study by D’Souza et al., it was shown that the risk of developing oral HPV infection increased with increases in lifetime oral or vaginal sex partners (*38*). It has also been reported that not only oral sex, but also open-mouthed kissing, was associated to the development of oral HPV infection (*38*). In this study, 2 study populations were included, one (332 patients) consisted of a control patient group >18 years of age from the Johns Hopkins outpatient otolaryngology clinic (2000–2006) enrolled in 2 case–control studies within a prospective cohort with HNSCC. The other (210 students) consisted of students >17 years of age recruited from the campuses of Towson University and the University of Maryland in 2007. The latter study may have had some limitations because it was not population based, and data for open-mouthed kissing for control patients and smoking for college students were absent. Nevertheless, this study suggests that oral-to-oral contact may play a role for oral HPV transmission and could play a major role in timing of prophylactic vaccination of children.

Several reports show an increase in OSCC and the proportion of HPV-positive OSCC and an association of the latter to early sex debut and many partners. Thus, we suggest that we are encountering a slow epidemic of mainly sexually transmitted HPV-induced OSCC.

## HPV in OSCC and Consequences for Treatment and Prevention

The possibility that we are dealing with an HPV-induced epidemic of OSCC warrants special attention. For example, in Stockholm, the incidence of HPV-positive tonsillar cancer has increased 7-fold over 30 years (*18*). OSCC now accounts for approximately one third of all HNSCC cases annually in Sweden. The fact that HNSCC in general is decreasing, and OSCC is increasing, may in 10 years result in OSCC accounting for half of all HNSCCs in Sweden, and similar trends are likely elsewhere, e.g., the United States, United Kingdom, the Netherlands, and Finland. It is also known that patients with HPV-positive OSCC are younger and have a better prognosis than HNSCC patients and patients with HPV-negative OSCC (*7**,**8*). In contrast, because of the poor prognosis for HNSCC and, in the past for OSCC, therapeutic measures have recently been intensified with induction chemotherapy, hyperfractionated radiotherapy, surgery, and occasional use of epidermal growth factor receptor inhibitors. This intensified therapy results in more severe acute and chronic side effects, such as difficulties in swallowing or talking, dry mouth, and necrosis of the jawbone, and is also more expensive for society. Accordingly, it is possible that increasing numbers of OSCC patients with a better prognosis are being treated with intensified therapy. As a result, many patients have substantial chronic unnecessary side effects. It is therefore necessary to identify which patients need and which do not need intensified treatment, both to increase patient survival times and quality of life and for the socioeconomic benefit of society.

Several reports have been published and other studies are ongoing to assess which molecular factors, such as p16, p53, and others, besides the presence of HPV in OSCC, can best predict clinical outcome and which treatments are optimal according to the same predictive markers (*7**–**9**,**17**,**26*). Some retrospective reports have suggested that persons with HPV-positive OSCC have higher response rates to chemotherapy and radiation; however, in other reports this has not been confirmed (*9**,**17*). A recent study also observed that tumor HPV status is a strong independent prognostic factor for survival among patients with HPV-positive OSCC irrespective of treatment (*16*). However, in the same study, among patients with HPV-positive tumors, the risk for death significantly increased with each additional pack-year of smoking, independent of treatment modality (*16*), a result similar to that found by Lindquist et al. (*13*).

It has been shown in an experimental setting that HPV-positive tumors were not more curable on the basis of increased epithelial sensitivity to cisplatin or radiation therapy (*39*). Instead, Spanos et al. demonstrated that radiation and cisplatin induced an immune response to this antigenic type of cancer. This finding could suggest that the presence of HPV in a tumor induced by smoking could be of benefit, but it is possible that smoking also may abrogate the immune response. As mentioned, the relationship between smoking and HPV and their roles in OSCC is most likely complex. In future studies, it would therefore be valuable to obtain more molecular and immunologic information and to determine if it is a survival benefit to stop smoking during and after therapy.

Summarizing treatment of OSCC patients, it is obvious that additional information will be required before it will be possible to guide treatment decisions for the individual patient on the basis of HPV status. Nevertheless, there is accumulating evidence that HPV status and overexpression of p16, and having never smoked, is of benefit. Future prospective clinical studies, including diagnostics of HPV, molecular and immunologic profiles, history of smoking, cessation of smoking during therapy, and effects of different treatment modalities and their side effects on quality of life, will be of benefit for personalized treatment.

Finally, it is also essential to keep in mind that we now have vaccines directed against HPV16, which accounts for ≈80%–90% of all HPV-positive OSCC, at least in Europe and the United States (*7**–**9**,**11**–**19*). Although it will likely take several decades before the effects of HPV vaccination on cancer incidence will be detected, it is crucial to monitor the effects of the present HPV vaccination, not only on the incidence of cervical cancer but also on the incidence of OSCC.

Few if any of other studies have focused on performing isolated health economic analysis of the effect of HPV on OSCC. However, a recent study pointed out that there is an improvement of the present cost-effectiveness of HPV vaccines when the effects on other HPV-associated tumors cancers are included (*40*). Furthermore, in countries with effective cervical cancer screening programs, other HPV-associated noncervical cancers represent a relatively high proportion of HPV-positive cancers (*15*). Considering that OSCC is the second most common HPV-associated cancer and its incidence is increasing, the effect of the HPV vaccine on this tumor deserves attention, and we need to know if future vaccination against HPV infection should include both women and men.
